# Validated Alzheimer’s Disease Risk Index (ANU-ADRI) is associated with smaller volumes in the default mode network in the early 60s

**DOI:** 10.1007/s11682-017-9789-5

**Published:** 2017-12-14

**Authors:** Nicolas Cherbuin, Marnie E. Shaw, Erin Walsh, Perminder Sachdev, Kaarin J. Anstey

**Affiliations:** 1 Centre for Research on Ageing, Health and Wellbeing, Australian National University, 54 Mills Road, Canberra, ACT 2601 Australia; 2 School of Psychiatry, University of New South Wales, Sydney, Australia

**Keywords:** Dementia risk, ANU-ADRI, DMN, MRI, MCI

## Abstract

Strong evidence is available suggesting that effective reduction of exposure to demonstrated modifiable risk factors in mid-life or before could significantly decrease the incidence of Alzheimer’s disease (AD) and delay its onset. A key ingredient to achieving this goal is the reliable identification of individuals at risk well before they develop clinical symptoms. The aim of this study was to provide further neuroimaging evidence of the effectiveness of a validated tool, the ANU Alzheimer’s Disease Risk Index, for the assessment of future risk of cognitive decline. Participants were 461 (60–64 years, 48% female) community-living individuals free of dementia at baseline. Associations between risk estimates obtained with the ANU-ADRI, total and regional brain volumes including in the default mode network (DMN) measured at the same assessment and diagnosis of MCI/dementia over a 12-year follow-up were tested in a large sample of community-living individuals free of dementia at baseline. Higher risk estimates on the ANU-ADRI were associated with lower cortical gray matter and particularly in the DMN. Importantly, difference in participants with high and low risk scores explained 7–9% of the observed difference in gray matter volume. In this sample, every one additional risk point on the ANU-ADRI was associated with an 8% increased risk of developing MCI/dementia over a 12-year follow-up and this association was partly mediated by a sub-region of the DMN. Risk of cognitive decline assessed with a validated instrument is associated with gray matter volume, particularly in the DMN, a region known to be implicated in the pathological process of the disease.

## Introduction

In the context of population ageing, Alzheimer’s disease (AD) is becoming an unsustainable burden at the individual, social and economic levels. Although investment in dementia research has increased very substantially in recent times, current evidence suggests that a disease modifying treatment is unlikely to be available in the clinic before a decade or more. Moreover, if and when such treatment becomes available, due to the multi-factorial nature of the disease, it will be unlikely to fully cure the complex underlying pathology or to be widely available or affordable in a timely fashion. In addition, it is now increasingly recognised that individuals in the pre-symptomatic stages and who have not already suffered extensive neurodegeneration are likely to benefit most from any pharmacological or non-pharmacological treatment.

Consequently, it is essential to develop strategies to identify cognitively healthy individuals who are at particular risk of developing AD in order to better target future treatments as they become available, and in the meantime, to implement preventative strategies aimed at decreasing risk in the population. This is particularly relevant as evidence confirming the contribution of modifiable risk factors to the disease process, and the effectiveness of interventions aimed at decreasing risk exposure is becoming increasingly strong (Xu et al. 2015).

With this in mind, we have developed a reliable evidence-based AD risk assessment instrument, called the Australian National University Alzheimer Disease Risk Index (ANU-ADRI), that does not require complex or costly clinical tests and which can be administered face-to-face or online (Anstey et al. 2013). It relies on robust estimates from high-quality published systematic reviews summarising the effect of fifteen established AD risk factors including age, sex, education, diabetes, body mass index (BMI), hypercholesterolemia, stroke, traumatic brain injury, depression, physical activity, smoking, dietary fish intake, alcohol consumption, pesticide exposure, cognitive and social engagement. Importantly, the ANU-ADRI has been externally validated in three large international cohort studies and was found to be reliable in predicting prospectively the development of Alzheimer’s disease, and was also found to be robust to the inclusion of an incomplete set of risk measures if all are not available (Anstey et al. 2014). In a recent study, we also demonstrated that higher scores on the ANU-ADRI were predictive of conversion to mild cognitive impairment (MCI) (Andrews et al. 2017).

The aim of the present study was to further build the evidence supporting the validity of this instrument by demonstrating a cross-sectional association between ANU-ADRI scores and individual variation in total brain volume (TBV), cortical gray matter (GM) volume, hippocampal volume (HC), and the volume of the structures of the brain default mode network (DMN). The DMN is particularly relevant in this context because it has been shown to be consistently affected by AD-related neurodegeneration relatively early in the disease process (Simic et al. 2014; Petrella et al. 2011; Fjell et al. 2014; Mormino et al. 1991). For example, amyloid plaque deposition assessed with PIB-PET is higher in the DMN and occurs concurrently to hypometabolism which also develops preferentially in this region (Grothe and Teipel 2016). The DMN is also one of the brain networks most affected by gray matter atrophy (Grothe and Teipel 2016; Tondelli et al. 2012). Importantly, these changes in the DMN are detectable well before (10 years or more) clinical diagnoses of MCI or AD are established in affected individuals (Tondelli et al. 2012; Sheline et al. 2010).

Thus, we predicted that higher ANU-ADRI scores in individuals participating in a large community-based study of ageing would be associated with lower total and region of interest (ROI) volumes. In addition, we hypothesised that risk scores and brain volumes related to risk levels would be predictive of conversion to MCI/dementia over a 12 year follow-up.

## Methods

### Study population

Participants included in the present study were selected from the larger PATH Through Life (PATH) project which has been described elsewhere (Anstey et al. 2012). Briefly, PATH randomly sampled individuals from the electoral roll of the city of Canberra and adjoining town of Queanbeyan across three age groups. The focus of this investigation is on the older cohort (*n* = 2551) aged 60–64 at baseline in 2001. Participants were included if they had undergone a brain scan at the first assessment (*n* = 551, wave 1) and met inclusion criteria. After excluding those with a MMSE < 25 (*n* = 29), stroke (*n* = 14), epilepsy (*n* = 2), or Parkinson’s disease (*n* = 10), 496 participants were available for analyses. A further 35 participants were excluded due to failed neuroimaging processing quality control criteria leaving 461 for further analysis. Compared to those excluded, included participants did not differ in age and sex but had a somewhat higher level of education (13.99 vs 13.71 years) and higher scores on the MMSE (29.34 vs 29.04).

### Standard protocol approvals, registrations, and patient consent

The study was approved by the Australian National University Ethics Committee and all participants provided written informed consent.

### Socio-demographic and health measures

Total years of education, stroke, depression symptomatology (Goldberg depression) (Goldberg et al. 1988), and smoking (ever) were assessed by self-report. Body mass index (BMI) was computed with the formula weight (kg)/height x height (m^2^) based on self-report of weight and height. Systolic and diastolic blood pressures were computed over two measurements using an Omron M4 monitor after a rest of at least 5 min. Participants were classified as hypertensive if their mean systolic or diastolic blood pressure measures were higher than 140 and 90 mmHg respectively or if they took anti-hypertensive medication. APO*E4 genotype was determined based on buccal swabs using QIAGEN DNA Blood kits (#51162; QIAGEN, Hilden, Germany). Participants were classified as APO*E4 carriers if they possessed one or two ɛ4 alleles and as non-carriers otherwise.

### MRI scan acquisition and image analysis

All participants were imaged with a 1.5 T Philips Gyroscan ACS-NT scanner (Philips Medical Systems, Best, The Netherlands) for T1-weighted 3-D structural scan (fast-field echo sequence [TR/TE/FA = 28.05/2.64 ms/30º] matrix size = 256 × 256; field of view = 260, for an in plane resolution of 1 mm × 1 mm). All images were pre-processed using the MINC imaging toolbox (MINC; http://en.wikibooks.org/wiki/MINC) which included image intensity normalisation and B_0_ inhomogeneity correction (Sled et al. 1998). Further image analysis was carried out using FreeSurfer (v. 5.3) (Fischl 2012). The image processing steps consist of motion correction and averaging, removal of non-brain tissue using a hybrid watershed/surface deformation procedure, automated Talairach transformation, and segmentation of the subcortical white matter and deep grey matter volumetric structures, intensity correction and delineation of grey/white/cerebrospinal fluid boundaries. Following completion of cortical models a number of deformable procedures were applied including surface inflation, registration to a spherical atlas (Dale et al. 1999) and parcellation of the cerebral cortex into neuroanatomical units based on gyral and sulcal structure (Desikan et al. 2006). The segmentation and parcellation processes use probabilistic information estimated from the manually labelled training set to assign a neuroanatomical label automatically to each voxel.

### Regions of interest

**Fig. 1:**
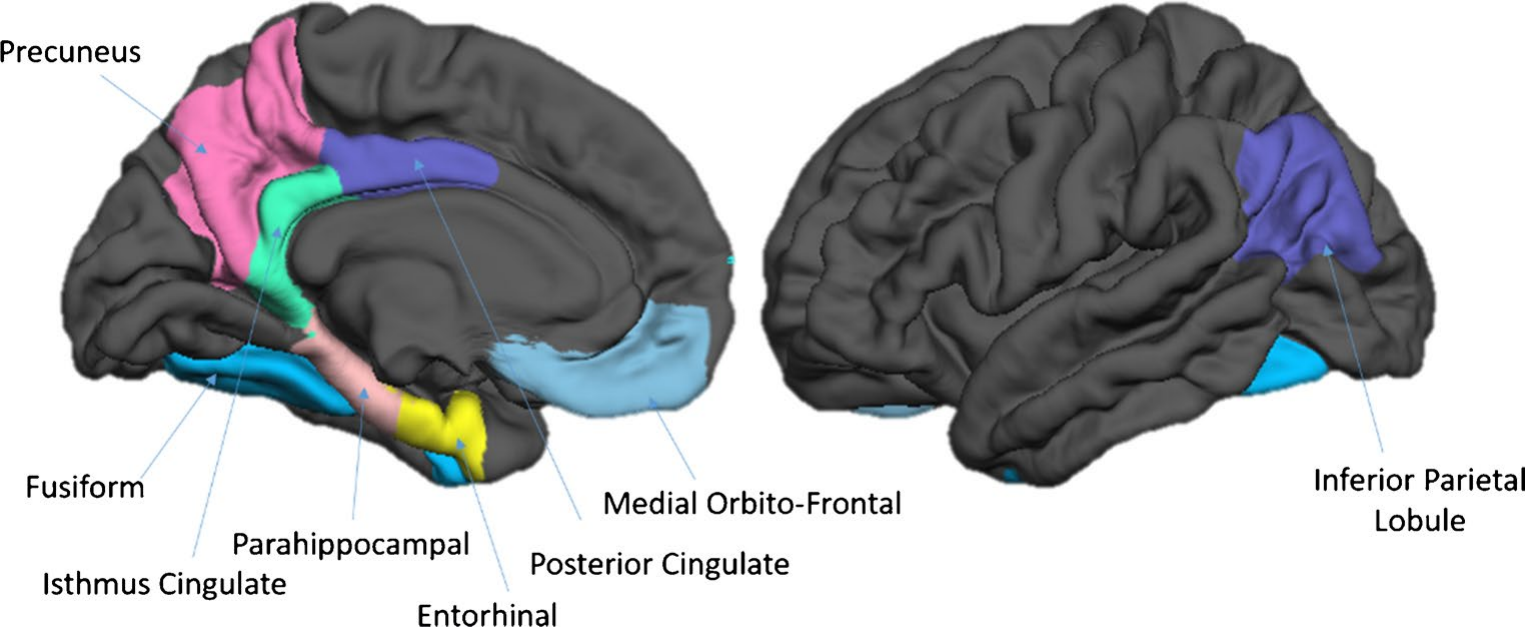
Sub-regions of the Default Mode Network (DMN)

Total brain volume (TBV) and cortical GM volumes were considered as global measures of cerebral health while HC and DMN were considered as specific ROIs known to be implicated in the AD pathological process (Simic et al. 2014; Grothe and Teipel 2016; Chang et al. 2015; Koch et al. 1991). The volumes of structures of the left and right DMN were computed by summing the volumes of their sub-components (see (Buckner et al. 2008; Laird et al. 2009; Andrews-Hanna et al. 2010; Thomas Yeo et al. 2011) for a rationale of this selection) including the Medial Orbito-Frontal (MOF), Entorhinal (ERC), Para-Hippocampal (P-HC), Fusiform (FUS), Posterior Cingulate (PCC), Isthmus Cingulate (ICC), PreCuneus (PCU) cortices and the Inferior Parietal Lobule (IPL) (Fig. 1).

### ANU Alzheimer’s Disease Risk Index (ANU-ADRI)

The ANU-ADRI (http://anuadri.anu.edu.au) is a questionnaire-based instrument which has been developed to estimate future risk of developing AD. To ensure its robustness it has been constructed based on risk estimates from published meta-analyses of established risk factors which can be assessed by self-report. The methodology used in its development is discussed and published elsewhere (Anstey et al. 2013). Briefly, it assesses up to 15 domains (age, sex, education, BMI, depression, diabetes, cholesterol, smoking, traumatic brain injury, physical activity, cognitive activity, social engagement, alcohol intake, dietary fish intake, pesticide exposure) and produces corresponding risk sub-scores by allocating points (weighted relative to each risk factor’s effect size) for varying levels of each domain reported by individual users. An overall composite score is computed by summing all available sub-scores. The ANU-ADRI has been validated against three large international studies and was found to be reliable in estimating one’s risk of developing AD and to be robust when not all risk measures are available for its computation (Anstey et al. 2014). In the present dataset, 11 available risk measures were considered for analysis including age, sex, education, depression, diabetes, smoking, traumatic brain injury, physical activity, cognitive activity, social engagement, alcohol intake. Note that while a BMI measure was also available it was not included as current evidence from meta-analysis indicates a risk for this factor is only present in mid-life.

### Diagnosis and cognitive change

Participants’ cognitive status was assessed at the first wave of assessment and re-assessed approximately every 4 years over a follow-up of 12 years (waves 2–4). At waves 1 to 3 diagnoses were assessed in a two-step process. Participants were first assessed on a number of cognitive measures including the MMSE, immediate and delayed recall, symbol-digit substitution test, simple and complex reaction time, and Purdue pegboard) as part of the main survey. If they performed below specific thresholds [any one of the following (1) a MMSE score ≤ 25; (2) a score below the 5th percentile on immediate or delayed recall, or (3) a score below the 5th percentile on one of the following tests: symbol-digit substitution test or Purdue Pegboard with both hands or reaction time] they were administered a full neuropsychological assessment on which a consensus diagnosis was established based on published criteria for MCI (Petersen/Winblad) and dementia (DSM-IV) (Petersen et al. 1999; Winblad et al. 2004). At the fourth wave, because of the higher prevalence of MCI and dementia in this age group, all participants were administered a neuropsychological assessment and given a diagnosis if they met clinical criteria. As an additional measure of cognitive decline a change score was computed between the latest MMSE score and the baseline score to reflect change in general cognitive functioning over the follow-up period.

### Statistical analysis

Statistical analyses were computed in the R statistical package (version 3.1). Descriptive analyses were conducted using Chi-square tests for categorical data and t-tests to compare groups on continuous variables. Cross-sectional associations between ANU-ADRI risk scores and TBV, cortical GM, HC and DMN structures volume at first assessment were investigated with linear regression analyses controlling for sex, age, intra-cranial volume (ICV) and APO*E4 genotype. Follow-up analyses were conducted in each hemisphere to identify topographical differences. Variables which did not follow a normal distribution were transformed with an appropriate function (log). Multivariate outliers were excluded if their Mahalanobis distance was greater than 13.82 (*p* < 0.001), leading to a maximum of 4 participants being excluded for any single ROI. Cox proportional hazard ratios were computed to test the associations between ANU-ADRI score, DMN volume and risk of cognitive impairment (MCI/dementia) or change in MMSE score over the follow-up (R package “survival”) with time to diagnosis as time metric. Alpha was set at 0.05 with Bonferroni correction.

## Results

**Fig. 2:**
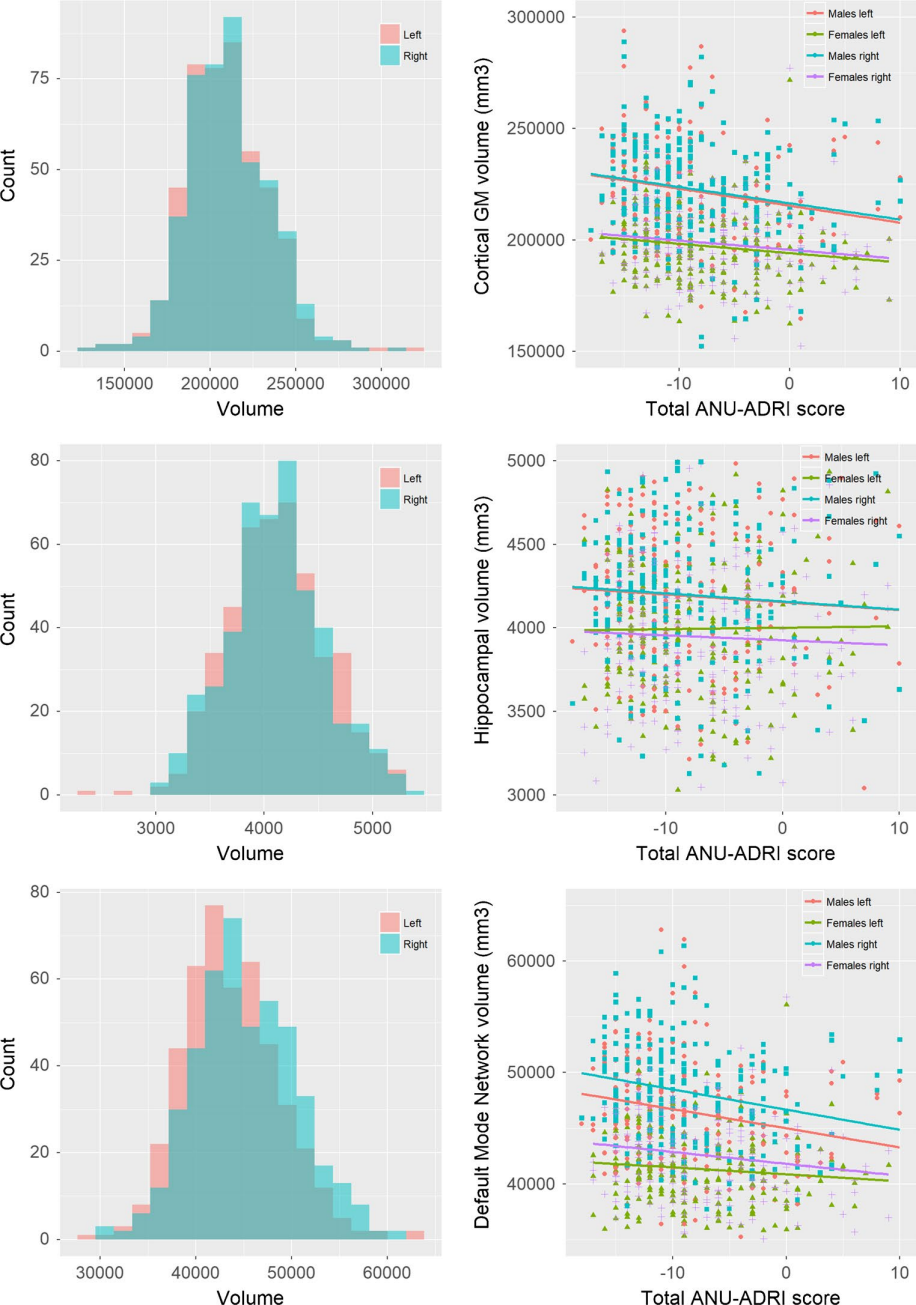
Distribution of left and right volume of structures in the Default Mode Network (left), and scatter plot of the association between ANU-ADRI total scores and volumes in the DMN (right)

The participants’ demographic measures are presented in Table 1. Male were not substantially different from female participants on most characteristics except form men being more likely to be hypertensive and for women having a slightly lower BMI and fewer years of education, as well as engaging somewhat less in physical and cognitive activity. The total ANU-ADRI scores ranged from − 18 to + 10 with a mean of − 8.20 (SD 5.67) indicating that on average participants were exposed to more protection than risk given their profile. Left GM (%) and HC (%) volumes were larger than right volumes. The volume of DMN structures was larger in the right than left hemisphere (~ 1%) and was composed of MOF (~ 11.2%), ERC (4.4%), P-HC (~ 4.8%), FUS (~ 20.9%), PCC (~ 6.7%), ICC (~ 5.5%), IPL (~ 27.3%), and PCU (19.2%) with each contributing a similar proportion of variance to the overall volume in the DMN. Histogram, scatter, and boxplots showing the distribution of left and right cortical gray matter, hippocampal and DMN structure volumes and their association with the total ANU-ADRI score are presented in Fig. 2.

**Table 1:** Participants’ demographic characteristics

Measures	Whole sample (*n* = 461)	Males (*n* = 238)	Females (*n* = 223)	T/chi-sq test (P value)
Age, years (SD)	63.05 (1.44)	63.10 (1.42)	63.00 (1.47)	0.78 (0.437)
Education, years (SD)	13.99 (2.62)	14.39 (2.57)	13.56 (2.60)	3.44 (0.001)
MMSE, score (SD)	29.34 (0.90)	29.29 (0.92)	29.41 (0.87)	− 1.46 (0.144)
BMI, kg/m2 (SD)	26.53 (4.31)	26.58 (3.47)	26.47 (5.07)	0.27 (0.791)
Hypertension, n (%)	285 (61.82%)	160 (67.23%)	125 (56.05%)	5.63 (0.018)
APO*E4, n (%)	123 (26.68%)	65 (27.31%)	58 (26.01%)	0.04 (0.833)
Alcohol, score (SD)	− 2.48 (1.14)	− 2.55 (1.08)	− 2.41 (1.20)	− 1.30 (0.194)
Education, score (SD)	0.61 (1.30)	0.50 (1.16)	0.73 (1.44)	− 1.82 (0.069)
Cognitive activity, score (SD)	− 4.92 (2.86)	− 5.26 (2.62)	− 4.56 (3.05)	− 2.65 (0.008)
Depression, score (SD)	0.05 (0.32)	0.03 (0.26)	0.07 (0.37)	− 1.27 (0.205)
Diabetes, score (SD)	0.23 (0.81)	0.26 (0.85)	0.20 (0.75)	0.84 (0.401)
Education, score (SD)	0.61 (1.30)	0.50 (1.16)	0.73 (1.44)	− 1.82 (0.069)
Physical activity, score (SD)	− 1.06 (1.17)	− 1.22 (1.19)	− 0.89 (1.13)	− 3.05 (0.002)
Smoking, score (SD)	0.64 (1.06)	0.70 (1.01)	0.57 (1.10)	1.29 (0.197)
Social engagement, score (SD)	0.72 (1.51)	0.59 (1.37)	0.85 (1.64)	− 1.86 (0.063)
TBI, score (SD)	0.17 (0.82)	0.27 (1.00)	0.07 (0.53)	2.66 (0.008)
ANU-ADRI, score (SD)	− 8.20 (5.67)	− 8.69 (5.65)	− 7.68 (5.66)	− 1.93 (0.054)
TBV, mm^3^ (SD)	1150449.66 (126363.06)	1222160.89 (115338.48)	1073914.81 (86528.32)	15.67 (0.000)
Left GM, mm^3^ (SD)	209832.88 (24694.96)	221804.07 (24817.49)	197056.46 (16985.34)	12.56 (0.000)
Right GM, mm^3^ (SD)	210880.75 (23986.38)	222450.90 (23761.95)	198532.35 (17108.24)	12.46 (0.000)
Left DMN, mm^3^ (SD)	35339.57 (4140.20)	37413.33 (3993.63)	33126.31 (2993.35)	13.09 (0.000)
Right DMN, mm^3^ (SD)	36531.44 (4360.38)	38696.23 (4186.08)	34221.04 (3209.80)	12.93 (0.000)
Left MOF, mm^3^ (SD)	5057.98 (772.34)	5382.60 (788.12)	4711.53 (582.54)	10.44 (0.000)
Right MOF, mm^3^ (SD)	4910.17 (654.92)	5176.12 (651.75)	4626.32 (527.74)	9.98 (0.000)
Left ERC, mm^3^ (SD)	2044.63 (372.38)	2176.18 (371.29)	1904.23 (319.05)	8.45 (0.000)
Right ERC, mm^3^ (SD)	1833.20 (372.47)	1942.74 (387.51)	1716.29 (317.12)	6.88 (0.000)
Left P-HC, mm^3^ (SD)	2195.33 (363.03)	2282.86 (412.63)	2101.91 (272.65)	5.59 (0.000)
Right P-HC, mm^3^ (SD)	2089.20 (384.64)	2153.98 (397.00)	2020.06 (359.18)	3.80 (0.000)
Left FUS, mm^3^ (SD)	9478.72 (1384.42)	9959.11 (1369.72)	8966.01 (1207.05)	8.27 (0.000)
Right FUS, mm^3^ (SD)	9107.03 (1393.85)	9681.33 (1374.17)	8494.11 (1130.83)	10.15 (0.000)
Left PCC, mm^3^ (SD)	2952.70 (505.92)	3148.05 (510.80)	2744.21 (408.93)	9.40 (0.000)
Right PCC, mm^3^ (SD)	3027.73 (515.74)	3196.79 (515.06)	2847.29 (452.33)	7.75 (0.000)
Left ICC, mm^3^ (SD)	2512.00 (453.11)	2681.97 (480.33)	2330.61 (339.25)	9.12 (0.000)
Right ICC, mm^3^ (SD)	2340.64 (411.27)	2435.37 (427.60)	2239.52 (367.95)	5.28 (0.000)
Left IPL, mm^3^ (SD)	11098.21 (1741.79)	11782.56 (1777.59)	10367.82 (1369.32)	9.61 (0.000)
Right IPL, mm^3^ (SD)	13223.48 (2057.40)	14109.90 (2059.66)	12277.43 (1581.33)	10.75 (0.000)

Scoring of the ANU-ADRI sub-components (i.e. alcohol, cognitive activity, depression, diabetes, education, physical activity, smoking, social engagement, traumatic brain injury) is presented in detail in (Anstey 2013)

*TBI* traumatic brain injury, *TBV* total brain volume, *GM* gray matter, *WM* white matter, *DMN* default mode network, *MOF* medial orbito-frontal, *ERC* entorhinal, *P-HC* para-hippocampal, *FUS* fusiform, *PCC* posterior cingulate, *ICC* isthmus cingulate, *PCU* precuneus cortices, *IPL* inferior parietal lobule

Significance: *p* < 0.05

### Associations between ANU-ADRI scores and DMN volume

Analyses investigating the relationship between ANU-ADRI scores and ROIs while controlling for age, sex, ICV and APO*E4 genotype revealed significant negative association with left and right cortical gray matter, and left and right DMN (Table 2). This indicated that for every one point increase in the ANU-ADRI there was an approximately 0.25% and 0.32% decrease in cortical and DMN structure volume respectively, which translates to an 7–9% difference in volume in these regions between those with the lowest and highest risk scores. No significant associations were detected with TBV, left or right hippocampus.

**Table 2:** Associations between ANU-ADRI scores and ROIs volumes assessed by multiple linear regression analyses and controlling for age, sex, ICV and APO*E4 genotype. Significance levels are reported after Bonferoni corrections

	TBV	Left GM	Right GM	Left HC	Right HC	Left DMN	Right DMN
ANU-ADRI	246.29	− 542.80^***^	− 557.86^***^	− 0.92	− 4.46	− 131.41^***^	− 162.19^***^
	*p* = 0.525	*p* < 0.001	*p* < 0.001	*p* = 0.782	*p* = 0.170	*p* < 0.001	*p* < 0.001
Sex	− 23,809.82^***^	− 10,914.52^***^	− 10,423.89^***^	− 70.98	− 116.21^**^	− 2,184.91^***^	− 2,446.86^***^
	*p* < 0.001	*p* < 0.001	*p* < 0.001	*p* = 0.111	*p* = 0.008	*p* < 0.001	*p* < 0.001
Age	− 4,589.17^**^	− 1,545.40^**^	− 1,910.45^***^	− 37.50^**^	− 32.83^*^	− 306.71^**^	− 396.22^**^
	*p* = 0.003	*p* = 0.005	*p* < 0.001	*p* = 0.005	*p* = 0.011	*p* = 0.009	*p* = 0.002
ICV	0.62^***^	0.06^***^	0.06^***^	0.01^***^	0.01^***^	0.01^***^	0.01^***^
	*p* < 0.001	*p* < 0.001	*p* < 0.001	*p* < 0.001	*p* < 0.001	*p* < 0.001	*p* < 0.001
APO*E4	3,333.17	522.79	1,412.52	19.20	14.57	177.26	397.37
	*p* = 0.499	*p* = 0.770	*p* = 0.424	*p* = 0.651	*p* = 0.725	*p* = 0.638	*p* = 0.316
Constant	486,015.10^***^	209,495.20^***^	233,341.60^***^	5,201.03^***^	4,792.94^***^	41,687.87^***^	47,935.97^***^
	*p* < 0.001	*p* < 0.001	*p* < 0.001	*p* < 0.001	*p* < 0.001	*p* < 0.001	*p* < 0.001
Adjusted R^2^	0.856	0.482	0.484	0.173	0.228	0.489	0.498
Residual Std. error	46,624.82	16,871.34	16,698.09	401.010	391.94	3,558.79	3,752.08
F statistic	544.57^***^	86.03^***^	86.67^***^	20.13^***^	28.16^***^	88.78^***^	92.02^***^
	(df = 5; 454)	(df = 5; 452)	(df = 5; 452)	(df = 5; 452)	(df = 5; 454)	(df = 5; 454)	(df = 5; 454)

Significance: *p < 0.05, **p < 0.01, ***p < 0.001

Follow-up analyses testing regional differences within the left and right DMNs are presented in Table 3. Significant associations were detected between ANU-ADRI scores and the P-HC, FUS, ICC, and IPL in the two hemispheres and with the left MOF. Trends were also detected for right MOF and PCC.

**Table 3:** Associations between ANU-ADRI scores and DMN sub-region volumes assessed by multiple linear regression analyses and controlling for age, sex, ICV and APO*E4 genotype. Significance levels are reported after Bonferoni corrections

ROI																
	MOF		ECC		P-HC		FUS		PCC		ICC		IPL		PCU	
	Left	Right	Left	Right	Left	Right	Left	Right	Left	Right	Left	Right	Left	Right	Left	Right
ANU-ADRI	− 15.64	− 8.45	− 3.54	− 2.78	− 6.31	− 6.50	− 43.71	− 42.78	− 4.23	− 7.41	− 7.31	− 7.70	− 29.39	− 50.92	− 27.2	− 30.64
	*p* = 0.002	*p* = 0.056	*p* = 0.199	*p* = 0.315	*p* = 0.011	*p* = 0.008	*p* < 0.001	*p* < 0.001	*p* = 0.244	*p* = 0.051	*p* = 0.019	*p* = 0.014	*p* = 0.012	*p* < 0.001	*p* = 0.001	*p* < 0.001
Sex	− 300.06	− 319.24	− 159.67	− 114.15	− 30.49	15.89	− 474.23	− 515.79	− 238.59	− 184.72	− 154.29	− 83.16	− 607.96	− 841.46	− 182.48	− 412.92
	*p* < 0.001	*p* < 0.001	*p* < 0.001	*p* = 0.002	*p* = 0.348	*p* = 0.627	*p* < 0.001	*p* < 0.001	*p* < 0.001	*p* < 0.001	*p* < 0.001	*p* = 0.047	*p* < 0.001	*p* < 0.001	*p* = 0.083	*p* < 0.001
Age	− 26.25	− 26.88	5.45	4.46	− 17.18	− 12.22	− 56.10	− 22.17	− 12.57	− 33.43	− 30.08^*^	− 17.37	− 105.59	− 198.84	− 87.06	− 84.45
	*p* = 0.179	*p* = 0.117	*p* = 0.608	*p* = 0.677	*p* = 0.073	*p* = 0.205	*p* = 0.154	*p* = 0.548	*p* = 0.367	*p* = 0.024	*p* = 0.013	*p* = 0.154	*p* = 0.021	*p* < 0.001	*p* = 0.005	*p* = 0.011
ICV	0.002	0.001	0.0005	0.0004	0.001	0.001	0.002	0.003	0.001	0.001	0.001	0.001	0.004	0.004	0.003	0.003
	*p* < 0.001	*p* < 0.001	*p* < 0.001	*p* < 0.001	*p* < 0.001	*p* < 0.001	*p* < 0.001	*p* < 0.001	*p* < 0.001	*p* < 0.001	*p* < 0.001	*p* < 0.001	*p* < 0.001	*p* < 0.001	*p* < 0.001	*p* < 0.001
APO*E4	167.87	0.131	13.69	20.57	12.94	− 10.20	98.56	192.16	12.86	− 73.02	− 42.10	16.62	− 33.40	80.43	− 19.61	150.75
	*p* = 0.009	*p* = 0.999	*p* = 0.693	*p* = 0.555	*p* = 0.677	*p* = 0.744	*p* = 0.441	*p* = 0.111	*p* = 0.777	*p* = 0.128	*p* = 0.282	*p* = 0.674	*p* = 0.821	*p* = 0.638	*p* = 0.845	*p* = 0.157
Constant	4,163.24	4,882.05	977.08	911.54	2,270.26	1,668.88	9,214.95	5,666.56	2,663.05	3,901.43	3,094.82	2,493.38	12,198.5	18,692.60	9,034.41	9,670.33
	*p* = 0.002	*p* < 0.001	*p* = 0.156	*p* = 0.188	*p* < 0.001	*p* = 0.008	*p* < 0.001	*p* = 0.018	*p* = 0.004	*p* < 0.001	*p* < 0.001	*p* = 0.002	*p* < 0.001	*p* < 0.001	*p* < 0.001	*p* < 0.001
Adj. R^2^	0.339	0.293	0.178	0.118	0.170	0.181	0.236	0.333	0.222	0.205	0.267	0.132	0.299	0.364	0.331	0.327
Res. Std. error	599.24	527.03	327.72	327.65	293.33	294.23	1,210.10	1,136.56	427.81	453.39	369.18	374.05	1,395.24	1,614.73	947.24	1,007.58
F statistic	47.888^***^	38.924^***^	20.860^***^	13.213^***^	19.813^***^	21.113^***^	29.415^***^	46.772^***^	26.987^***^	24.705^***^	34.304^***^	14.915^***^	40.069^***^	53.474^***^	46.277^***^	45.536^***^
N	459	458	460	457	459	457	461	459	457	460	458	459	459	459	458	460

Significance: *p < 0.05, **p < 0.01, ***p < 0.001

### ANU-ADRI score, DMN, and risk of cognitive impairment

Although the ANU-ADRI has been validated in three large international datasets and its association with increased risk of MCI has been demonstrated within the larger PATH cohort (Andrews et al. 2017), it was deemed important to confirm its predictive value for MCI/dementia within the sample studied here. Of the participants included at baseline and without any cognitive impairment 3 participants developed dementia (2 of those were previously diagnosed with MCI), 51 developed MCI (including the two who were subsequently diagnosed with dementia), and 409 remained cognitively healthy over the 12-year follow-up. Cox proportional hazard ratio analyses revealed that the ANU-ADRI score was significantly associated with conversion to MCI/dementia (HR 1.08, 95%CI 1.03–1.13, *p* = 0.002) after controlling for age, sex and APO*E4 genotype. Of the five DMN ROIs (volumes transformed into ml to facilitate interpretation) which were significantly predicted by the ANU-ADRI score, the left MOF was the only region found to be associated with an increased risk of developing MCI/dementia (HR 0.61, 95%CI 0.04–0.92, *p* = 0.018). This indicates that for every additional 1 ml in MOF volume at baseline the risk of cognitive impairment over the follow-up decreased by 64%. Finally, a formal mediation analysis (Baron and Kenny 1986) was conducted to determine whether the risk of developing MCI/dementia associated with a higher ANU-ADRI score was mediated by left MOF volume. It revealed a partial mediation as the HR and significance associated with the ANU-ADRI score (HR 1.07, 95%CI 1.02–1.13, *p* = 0.005) were significantly reduced (Chi square = 7.27, *p* = 0.03) when MOF volume was controlled for in the analysis. None of the total left or right DMN and left or right GM volumes significantly predicted MCI/dementia (results not shown) and therefore mediation analyses for these structures could not be conducted.

The associations between ANU-ADRI score and baseline MMSE score and change in MMSE score over the follow-up were also tested and revealed significant effects (Baseline MMSE estimate: − 0.038, *p* = 0.007; MMSE change estimate: − 0.025, *p* = 0.012) indicating that those in the lower ANU-ADRI quartile (less risk) would be predicted to have a 0.53 point higher MMSE score at baseline and a 0.35 point lower decrease in MMSE score over the follow-up than those in the upper quartile (given 14 points difference between first and last ANU-ADRI quartiles).

### Sensitivity analyses

All analyses were controlled for APO*E4 genotype to ensure the effects demonstrated occur above and beyond the risk imparted by the main AD genetic risk factor. However, to determine whether significant shared variance explained part of the relationship between ANU-ADRI score, volumes of DMN structures, and cognitive functioning, all analyses were repeated without controlling for APO*E4 genotype. Results (not shown) remained almost identical suggesting that the risk indexed by the ANU-ADRI explains variance in outcome measures unrelated to APO*E4.

## Discussion

This study’s main findings are that greater exposure to risk factors associated with the development of AD, as assessed with the ANU-ADRI, in community living participants in their early 60s is associated with lower brain volumes (cortical GM and DMN), a greater risk of experiencing decline in general cognitive functioning later in life, and a greater risk of developing MCI.

Higher scores on the ANU-ADRI were associated with lower volumes in the left and right cortical GM and DMN. This effect was very substantial with every additional point being associated with a 0.32% lower volume of DMN structures which equates to a 9% difference in volume across the range of scores observed in this sample. In addition, risk scores were differentially associated with sub-regions of the DMN with effects being greatest for the left MOF (0.3%/pt), left and right P-HC (0.29/0.31%/pt), left and right FUS (0.46/0.47%/pt), left and right ICC (0.29/0.33%/pt), and left and right IPL (0.27/0.39%/pt). It is also noteworthy that all DMN sub-regions were negatively associated with the risk score whether significantly so or not.

Importantly, ANU-ADRI risk scores also predicted baseline general cognitive functioning, with each additional risk point being associated with a 0.14% lower MMSE score, as well as change in function (− 0.13%/pt for each additional year of follow-up). This is significant as for a person with a risk score of 5 (compared to a score of 0 with no risk and no protection) it would correspond to a 6.5% lower MMSE over a 10 year period. Consistent with these findings results also showed that higher risk scores were associated with an 8% increased risk of developing MCI/dementia over the 12-year follow-up.

To test the hypothesis that DMN structures mediate the relationship between risk scores and cognitive impairment further analyses investigating the association between DMN sub-regions shown to be significantly predicted by ANU-ADRI scores and risk of developing MCI/dementia were conducted. They revealed that for every 1% larger MOF volume there was a 3.2% decreased risk of developing MCI/dementia over the follow-up. Importantly, the subsequent mediation analysis showed that the effect linking ANU-ADRI risk score level and risk of developing MCI/dementia was partially mediated by the left MOF (but not not by total GM) and thus supports a theoretical link between risk exposure, brain structure in the DMN, and cognitive impairment.

Importantly, all analyses were controlled for APO*E4 genotype which could potentially explain both the difference in volume and the difference in cognition observed. Moreover, additional sensitivity analyses which did not control for APO*E4 genotype indicated that the present effect were neither partially masked nor explained by this AD genetic risk factor. In fact, somewhat surprisingly, APO*E4 status did not explain any of the outcome variable except for the left MOF where being a carrier was associated contrary to what might have been expected, with a 3.3% increased volume. This is consistent with our previous research showing that the ANU-ADRI is a stronger predictors of cognitive decline than a composite genetic index including APO*E4 and other AD risk genes (Andrews et al. 2017).

A question arising from these findings is whether the variability in DMN volume related to risk estimated with the ANU-ADRI is specific to AD pathology or more generally related to the vulnerability of the DMN to a range of assaults due to its high and sustained metabolism, even at rest, relative to other brain networks. On one hand it is clear that a number of factors contributing to the ANU-ADRI score are also associated with increased amyloid plaque formation which would suggest that risk estimates are relatively specific to AD pathology (Luciano et al. 2015; He et al. 2017; Johnson et al. 2010). On the other hand, these same factors are associated with broad pathological mechanisms including systemic inflammation, oxidative stress, atherosclerosis, and impaired cardio-metabolic function which may have a less specific impact on the DMN via small vessel disease, chronic hypo-perfusion, micro-infarcts, and shrinking neuronal processes. However, given that up to 80% of AD cases have comorbid vascular disease (Attems and Jellinger 2014) this distinction may be moot. Nevertheless, it would be interesting for future studies to investigate whether ANU-ADRI estimates are specifically related to incident amyloid plaque formation.

Overall, these findings are important because they show that exposure to modifiable risk factors, when assessed with a valid instrument, is significantly associated with lower volumes in a brain network known to be particularly strongly affected in AD thus confirming previously demonstrated associations between risk factor exposure and risk of cognitive impairment. As such this study adds to the evidence base necessary to recommend to individuals living in the community that they decrease their risk exposure. It also indicates that behavior optimization and targeted preventative interventions should occur at mid-life or preferably before as the pathological processes mediating the effect of modifiable risk factors are likely to develop progressively over decades. These findings are also encouraging because they suggest that identifying individuals at risk on a large scale in a population is feasible since the ANU-ADRI can be administered online and has already been implemented in a free web-portal accessible by all (http://anuadri.anu.edu.au). Thus, those involved in recruiting participants for behavioral or pharmacological studies may want to consider the ANU-ADRI as an effective and inexpensive screening method.

This research has many strengths including a large sample size for a neuroimaging study, both brain and cognitive outcomes, the use of a validated measure of risk exposure and clear theoretically based hypotheses. However, it also has a number of limitations. Relationships between ANU-ADRI risk scores and brain were estimated with cross-sectional analyses and therefore do not permit causal interpretations. The ANU-ADRI scores were computed based on a sub-set of risk factors and while our previous validation studies have shown that the measure was robust to inclusion of a variable number of risk factors, availability of all included risk factors may have provided more accurate estimates and more powerful predictions. Finally, this investigation focused on a narrow age range which has the benefit of reducing cohort effects but may produce findings not be completely applicable to other age groups.

In summary, the evidence on modifiable risk factors is strong, screening for individuals at risk is achievable in general, but is made much more accessible through instruments such as the ANU-ADRI. This and other confirmatory studies provide confidence on the relevance, validity, and usefulness of this tools. A stronger focus needs to be put on behavior change to achieve risk reduction in the population.
